# Clinical, Radiological, and Etiological Aspects of Pachymeningitis: A Study of 24 Cases

**DOI:** 10.7759/cureus.61988

**Published:** 2024-06-09

**Authors:** Yousfi Samah, Boulehoual Sahar, Yassine Mebrouk

**Affiliations:** 1 Department of Neurology, Mohamed VI University Hospital, Mohamed First University, Faculty of Medicine, Oujda, MAR

**Keywords:** sarcoidosis, cerebral tuberculosis, infiltration of the dura mater, mri, hypertrophic pachymeningitis

## Abstract

Introduction and importance

Hypertrophic pachymeningitis (HP) is an uncommon disorder with varied etiological origins and heterogeneous clinical presentation. Establishing the etiological diagnosis poses a challenge, but prompt identification provides a treatment window, potentially leading to a reversal of symptoms. MRI is the reference examination, allowing not only the early diagnosis of pachymeningitis but also the assessment of its extent and importance, detection of possible complications, and suggestion of etiology.

Case presentation

We conducted a retrospective study involving 24 patients recruited over 5 years for who brain imaging had revealed the presence of pachymeningitis. The average age of the patients was 40 years, with a male-to-female ratio of 0.6.

Clinical discussion

Headache was present in 54.17% of patients. All the patients underwent MRI examinations utilizing different sequences, with subsequent Gadolinium injection showing localized and asymmetrical meningeal thickening in 13 cases, and diffuse in the rest. The cerebrospinal fluid study unveiled an inflammatory fluid characterized by a lymphocytic predominance and hyperproteinorrhea, noted in 50% of the patients. The histopathological analysis of a stereotactic biopsy conducted on an individual patient revealed non-diagnostic results. The etiological investigation was dominated by tuberculosis, which was detected in 33.3% of cases. Idiopathic origin was identified in 16.7% of patients.

Conclusion

Meningeal thickening is rare, and the multitude of potential causes makes the etiological investigation challenging unless they fall within the scope of secondary meningeal disorders; otherwise, a dural biopsy becomes necessary, and the prompt initiation of treatment, along with determining the etiology influences the prognosis.

## Introduction

Pachymeningitis (PM) is defined by inflammatory and fibrous infiltration of the dura mater [1], it can manifest either cranially, affecting cranial nerves and causing headaches, or spinally, resulting in radicular or medullary compressions [2]. Notable clinical manifestations of PM include headache and involvement of cranial nerves such as the sixth cranial nerve, seventh nerve, eleventh pair, and optic nerve. Additionally, symptoms like intracranial hypertension, seizures, cerebral venous thrombosis, and cerebellar manifestations often prompt consideration for PM diagnosis [3].

Magnetic resonance imaging (MRI) serves as the cornerstone diagnostic tool, enabling early identification and severity assessment of PM. Furthermore, it aids in detecting potential complications, such as thrombosis of the superior longitudinal sinus or parenchymal extension, and provides insights into the underlying etiology [4].

Etiologies of PM are varied, including infectious agents like syphilis and tuberculosis, inflammatory conditions such as sarcoidosis and granulomatosis with polyangiitis, IgG4-related disease, as well as idiopathic origins. Meningeal biopsy is highly useful for etiological diagnosis [5].

Corticosteroids serve as the primary treatment modality for PM. However, the efficacy and appropriateness of immunosuppressive drugs or rituximab usage remain subject to determination and should be tailored to the specific etiology [3].

The intricate nature of managing and diagnosing pachymeningitis presents substantial challenges for neurologists. This study aims to confront these challenges by elucidating the diagnostic and therapeutic aspects through insights gained from a retrospective analysis of 24 patient cases.

## Materials and methods

We conducted a retrospective study on 24 patients followed at the neurology department at Mohammed VI University Hospital from February 2018 until December 2023. Data, including clinical, demographic, imaging, and follow-up data, were collected from the medical records of the neurology department.

The assessment of all patients has been conducted at the University Hospital, with imaging at the Radiology department equipped with a 1.5 Tesla MRI and a 64-slice computed tomography (CT). The imaging reports were interpreted by a resident and then supervised by at least one professor of radiology with over 10 years of experience in a university hospital.

All patients underwent a CT scan before and after the injection of contrast medium, followed by MRI in different weightings and after the injection of Gadolinium. In six cases, an additional spinal MRI was conducted. All patients underwent a comprehensive biological assessment along with regular follow-up.

Written consent was obtained from all patients, and retrospective analysis was deemed unnecessary for ethical review by the ethics committee.

## Results

Our study includes patients of both sexes with a female predominance in 62.5% of cases, resulting in a male-to-female sex ratio of 0.6 with a mean age of 40 years.

In our series, 8.3 % of patients had a history of neoplasms, 20% had a recent ENT infection, and a history of incompletely treated pulmonary tuberculosis was revealed in 8.3 % of patients.

The majority of cases presented with a subacute onset. The indicative clinical signs were diverse. The clinical manifestations varied, with a predominant association observed in different degrees: headaches (in 13 cases), cranial nerve paralysis (in seven cases), a confusional state (in two cases), reduced visual acuity (in one case), and seizures (in one case).

**Figure 1:**
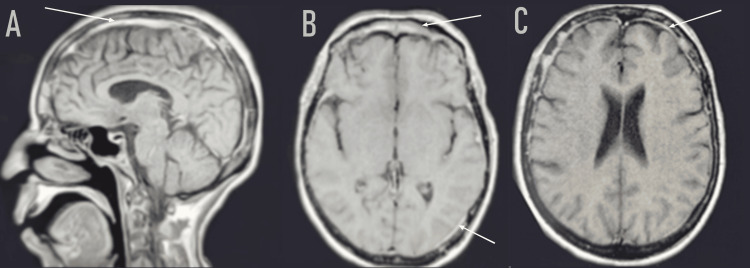
Brain MRI with T1-weighted sequence post-gadolinium injection, sagittal (A) and axial (B, C) sections revealing diffuse meningeal thickening (white arrows)

Cross-sectional imaging revealed a localized and asymmetrical meningeal thickening in 13 cases, and diffused in 11 cases, showing significant enhancement after contrast agent injection (Figure 1). Intraparenchymal-associated lesions were found in 17 cases. The cerebrospinal fluid study, conducted in all cases; revealed an inflammatory fluid characterized by a lymphocytic predominance and hyperproteinorrhea, in 50% of the patients, and biological abnormalities detected in 66.7% of patients, facilitated the determination of the etiology. Quantiferon was positive in 20.83%. GeneXpert confirmed TB involvement in 12.5%. The blood level of the angiotensin-converting enzyme is elevated by 12.5%. The labial biopsy performed in 90% of cases showed an epithelial-giganto-cellular granuloma without caseous necrosis in 8.33%.

A stereotactic brain lesion biopsy was performed on only one patient, and the histopathological study was inconclusive.

**Figure 2:**
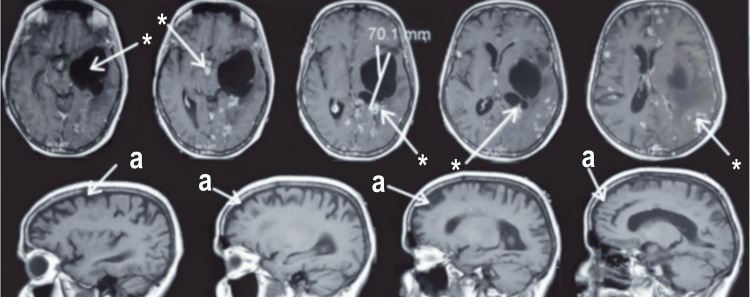
Brain MRI, axial section with T1-weighted sequence post-gadolinium injection, and sagittal T1-weighted sequence of a 47-year-old female patient with neurosarcoidosis associated with a left temporal 70 mm long-axis liquid lesion *: Diffuse micro and macronodular thickening of the meninges above and below the tentorium with a predominantly hemispheric involvement in the left periventricular and pericallosal areas, hyperintense on FLAIR, enhancing significantly after contrast administration. a: Pachymeningitidis FLAIR: fluid-attenuated inversion recovery

Pachymeningitis was secondary to tuberculosis (33.33%), Behçet's disease (12.5%), sarcoidosis (16.7%) (Figure 2), carcinomatous leptomeningitis (12.5%), and ENT infection (8, 33%). The remaining cases were found to be idiopathic (16, 7%).

A comprehensive summary of results for patients is presented in Table 1.

**Table 1:** Results for 24 patients ENT infection: ear, nose, and throat infection

Demographic data		
Male	9	37.5 %
Female	15	62.5 %
Sex ratio M/F	0,6	
Mean age	40 years ( from 19 to 78)	
Medical history		
Neoplasms	2	8.3%
ENT infection	5	20.83 %
Pulmonary tuberculosis	2	8.3%
Clinical manifestation		
Headaches	13	54.17%
Cranial nerve paralysis	7	29.17%
Confusional state	2	8.3 %
Reduced visual acuity	1	4.17%
Seizures	1	4.17 %
MRI imaging		
Localized and asymmetrical meningeal thickening	13	54.17%
Diffused meningeal thickening	11	45.83%
Intraparenchymal lesions	17	70.83 %
Biological parameters		
Cerebrospinal fluid study		
Lymphocytic predominance	12	50%
Hyperproteinorrhea	12	50%
Quantiferon	5	20.83%
GeneXpert	3	12.5%
Blood angiotensin-converting enzyme	3	12.5%
Labial biopsy		
Performed	21	90%
Results		
Granuloma without caseous necrosis	2	8.33%
Inconclusive	19	91.7 %
Stereotactic brain lesion biopsy	1	
Performed	1	4.17%
Result	Inconclusive	
Etiology		
Tuberculosis	8	33.33%
Behçet's disease	3	12.5%
Sarcoidosis	4	16.7%
Carcinomatous leptomeningitis	3	12.5%
ENT infection	2	8.33%
Idiopathic	4	16.7%

## Discussion

Cranial pachymeningitis is classified into two groups according to the site of dural thickening. One impacts the parasellar and cavernous regions, potentially reaching the cavernous and supra-clinoidal segments of the internal carotid artery and optic nerves. The other affects the posterior third of the scythe, cerebellar tent, and dura mater at the level of the clivus [5].

Symptoms are varied, depending on their etiologies. The most frequent clinical manifestations include headache and cranial nerve engagement, mainly the sixth cranial nerve when pachymeningitis affects the sphenoid sinus due to its proximity [6]. The eleventh pair is most often spared due to its slightly more caudal emergence from the brainstem. The optic nerve is frequently affected, occasionally on both sides. Intracranial hypertension, seizures, cerebral venous thrombosis, hearing impairment, and gait ataxia are often suggestive of the diagnosis [4,7].

The exploration of pachymeningitis relies on MRI, to a lesser extent on CT. The CT scan is much less effective than MRI in a positive diagnosis, especially in cases of minimal to moderate lesions, it reveals a hyperdense thickening of the dura mater, enhancing after contrast agent injection [8-9]. MRI is the reference examination. It not only enables the early diagnosis of pachymeningitis but also the evaluation of its importance and extent, detects potential complications (thrombosis of the superior longitudinal sinus or parenchymal extension), and suggests the etiology. The thickening of the dura mater appears as a hypointense signal on T1- and T2-weighted sequences and enhances significantly on post-contrast T1 sequences after gadolinium injection. In T2 sequences, the hypointense signal related to the density of fibrous tissue may be bordered by a thin peripheral strip of hypersignal indicating the hypervascularization of the injured dura mater. Diffuse hypersignal in T2 is also reported [4,10,11].

The use of biology, particularly the cytobacteriological and biochemical study of cerebrospinal fluid (CSF), is limited in its usefulness during the diagnostic process, except for infectious and tumoral etiologies. Hyperproteinorachia is frequent, as it is a lymphocytic reaction. Meningeal biopsy is of definite interest in etiology, especially when pachymeningitis is isolated. Although the constraint is that in some cases the fragment is minimal and difficult to interpret, it can reveal tumor cells, granulomatous lesions in syphilis, tuberculosis, sarcoidosis, granulomatosis with polyangiitis associated with caseous or fibrinoid necrosis, depending on the etiology [4,12].

Diagnosing the etiology of HP through clinical, biological, and imaging investigations remains a complex task, and the ultimate confirmation often relies on tissue biopsy. It is crucial to exclude the presence of infectious diseases. Particularly, syphilis, which was the first recorded cause of pachymeningitis [13]. Tuberculosis is also a classic cause of pachymeningitis [14]. Furthermore, otitis or complicated bacterial sinusitis can exhibit meningeal participation, resulting in hypertrophic pachymeningitis adjacent to the infectious process [15]. Fungal meningitis mainly caused by Aspergillus induces also thickening of the dura mater [16].

Inflammatory etiologies are dominated by sarcoidosis [17], immunoglobulin G4 (IgG4)-related disease [18], and granulomatosis with polyangiitis (GPA) [19,20].

Various neoplastic conditions can result in meningeal involvement, imitating hypertrophic pachymeningitis (HP). Secondary dural metastases may share similarities with HP [21]. Polyneuropathy organomegaly endocrinopathy monoclonal gammopathy skin changes syndrome (POEMS) has been described in association with cranial pachymeningitis [22].

The diagnosis of idiopathic pachymeningitis is established through the elimination of alternative causes of pachymeningitis and a negative biopsy. This condition specifically affects the pachymeninges, with its primary symptoms being headaches, visual disturbances, and ataxia [3].

The treatment depends on the underlying cause. For idiopathic hypertrophic pachymeningitis (IHP), first-line treatment typically involves corticosteroid therapy. If there is no improvement or if the condition recurs during corticosteroid administration, supplementation with immunosuppressive agents, such as azathioprine or cyclophosphamide, may be necessary, with adjustments based on lymphocyte nadir occurring 10 to 14 days later. Methotrexate (MTX), in combination with rituximab, is also considered a viable option [23-25].

## Conclusions

HP is a highly complex disorder with variable etiologies and heterogeneous clinical presentation. The etiology of HP is a challenge, despite a thorough clinical, laboratory, and imaging investigation. Tissue biopsy remains the gold standard for final diagnosis. Early clinical and etiological diagnosis is relevant to decide about therapy.
